# Animal Models for In Vivo Lactation Studies: Anatomy, Physiology and Milk Compositions in the Most Used Non-Clinical Species: A Contribution from the ConcePTION Project

**DOI:** 10.3390/ani11030714

**Published:** 2021-03-05

**Authors:** Domenico Ventrella, Nurit Ashkenazi, Alberto Elmi, Karel Allegaert, Camilla Aniballi, Anthony DeLise, Patrick John Devine, Anne Smits, Lilach Steiner, Monica Forni, Michele Bouisset-Leonard, Maria Laura Bacci

**Affiliations:** 1 Department of Veterinary Medical Sciences, University of Bologna, Ozzano dell’Emlia, 40064 Bologna, Italy; domenico.ventrella2@unibo.it (D.V.); camilla.aniballi2@unibo.it (C.A.); monica.forni@unibo.it (M.F.); marialaura.bacci@unibo.it (M.L.B.); 2 Global Research and Development, Teva Pharmaceutical Industries Ltd., Netanya 42504, Israel; Nurit.Ashkenazi01@teva.co.il (N.A.); Lilach.steiner@teva.co.il (L.S.); 3 Department of Development and Regeneration, KU Leuven, 3000 Leuven, Belgium; karel.allegaert@uzleuven.be (K.A.); anne.smits@uzleuven.be (A.S.); 4 Department of Clinical Pharmacology and Pharmacotherapy, KU Leuven, 3000 Leuven, Belgium; 5 Department of Hospital Pharmacy, Erasmus MC, 3015 GD Rotterdam, The Netherlands; 6 Novartis Pharmaceuticals Corporation, Novartis Institutes for BioMedical Research, East Hanover, NJ 07936, USA; anthony.delise@novartis.com; 7 Novartis Pharmaceuticals Corporation, Novartis Institutes for BioMedical Research, Cambridge, MA 02139, USA; patrick.devine@novartis.com; 8 Neonatal Intensive Care Unit, University Hospital Leuven, 3000 Leuven, Belgium; 9 Novartis Pharma AG, Novartis Institutes for BioMedical Research, 4056 Basel, Switzerland; michele-bouisset-leonard@novartis.com

**Keywords:** animal models, mammary gland, lactation, milk, colostrum, mice, rats, rabbits, dogs, non-human primates, pigs, minipigs, human

## Abstract

**Simple Summary:**

Nowadays, the importance of breastfeeding has been very well recognized not only by the scientific world but also by public opinion. Such awareness has nonetheless put a lot of pressure on women under chronic pharmacological medication, or that simply need to alleviate common post-partum health issues, due to the lack of scientific data regarding the potential transfer to the offspring during lactation. In such a scenario, the ConcePTION project aims at creating a trusted ecosystem that can efficiently generate and disseminate reliable evidence-based information regarding the effects of medications used during pregnancy and breastfeeding to women and their healthcare providers. Due to the need for a reliable animal species to obtain scientific data, the present review summarizes the main features contributing to the lactation process in the most commonly used laboratory animal species.

**Abstract:**

The present review aims to summarize the main features of mammary gland anatomy, and the physiology of lactation and colostrum/milk in the most commonly used animal species for regulatory toxicity. The final goal is the selection of a preferred animal species to be enrolled in studies investigating the potential transfer of drugs and exogenous molecules through milk, within the Innovative Medicines Initiative (IMI) funded project ConcePTION. Reference data regarding humans were also collected and analyzed in order to highlight critical similarities and differences with the studied species. Additional practical considerations were also taken into account, such as ethical consideration regarding the chosen species which affects the group size, financial implications and technical feasibility of lactation trials (e.g., ease of sampling, volume of sampling, husbandry requirements and scientific recognition). In conclusion, the present analysis of the literature confirms the complexity of the decisional process behind the choice of an animal model for in vivo trials. For some of the evaluated species, data were either poor or missing, highlighting the necessity to generate more physiological background studies for species that are routinely used in laboratory settings. Overall, when taking into consideration ethical factors, feasible group size, milk volume and ease of milk collection, and physiological similarities with humans, minipigs seem to represent the most appropriate choice.

## 1. Introduction

As of today, the importance of breastfeeding has been very well recognized not only by the scientific world but also by public opinion. Indeed, colostrum and breast milk represent the gold standard when it comes to nutritional and protective values [1], as also recommended by the World Health Organization [2]. Such awareness has nonetheless put a lot of pressure on women under chronic pharmacological medication, or that simply need to alleviate common post-partum health issues such as infections, mastitis, and headaches [3]. Indeed, the lack of reliable evidence-based knowledge regarding the safety of medications during lactation often leads physicians and medical practitioners to advise women to stop breastfeeding [4,5]. Behind the lack of scientific data are a plethora of different reasons, including the absence of a recognized “state of the art” animal model for preclinical studies aimed at testing the potential transfer through milk of systemically administered exogenous compounds such as drugs and medications.

Choosing the best animal model for a given experiment can be challenging and represents a complex decisional process that should take into account a wide variety of factors [6]. Generally speaking, the decision should be made upon in-depth consideration of: (i) analogy/homology; (ii) translation value to humans; (iii) genetic standardization; (iv) biological background knowledge; (v) cost/availability; and (vi) adaptability to experimental procedures [7]. For lactation studies, the key features to be analyzed are anatomy of the mammary gland, physiology of lactation, and colostrum/milk composition. Due to the pivotal role played by gastrointestinal/energetic metabolism in every biological process, ruminants should be used very cautiously when selecting an animal model for metabolism-related studies. Indeed, their peculiar gastrointestinal anatomy and physiology results in a relatively lower translational value when (drug) metabolism is involved, thus creating strong biases when considering the overall aim of such trials.

Literature searches were performed to identify a candidate non-clinical species relevant to humans for use in lactation studies and experimental trials. Species taken into account were the most commonly used in regulatory toxicology and included rodents (rat and mice), rabbits, dogs, pigs (both conventional and minipigs) and non-human primates (NHPs) [8]. Reference data regarding humans were also collected and analyzed in order to highlight critical similarities and differences with the studied species.

A preliminary scanning of the literature highlighted a relative lack of relevant data in the most used databases such as PubMED and EMBASE, making a systematic approach unfeasible. Indeed, basic data regarding anatomy and physiology are often found in textbooks or old papers and used only as reference/control data in more recent studies. Therefore, it was decided to broaden the literature search to currently used veterinary medicine textbooks, and to use less-specific search words and their combination for the different subcategories. Further literature searches were then performed starting from the reference sections of the retrieved articles.

The search criteria are provided below.

Anatomy of the mammary gland: preliminary search words included (“mammary gland” OR “udder”) AND (“anatomy” OR “morphology” OR “structure”) AND the aforementioned different species and their synonyms.

Physiology of lactation: preliminary search words included (“lactation” OR “milk production” OR “colostrum production”) AND (“physiology”) AND the aforementioned different species and their synonyms.

Colostrum/milk composition: preliminary search words included (“colostrum” OR “milk”) AND (“composition” OR “components” OR “quantitative composition”) AND the aforementioned different species and their synonyms.

Retrieved articles, book chapters and books were then analyzed to identify potential biases to the reported results as methodological errors or experimental design errors.

## 2. Anatomy of the Mammary Glands

**Figure 1:**
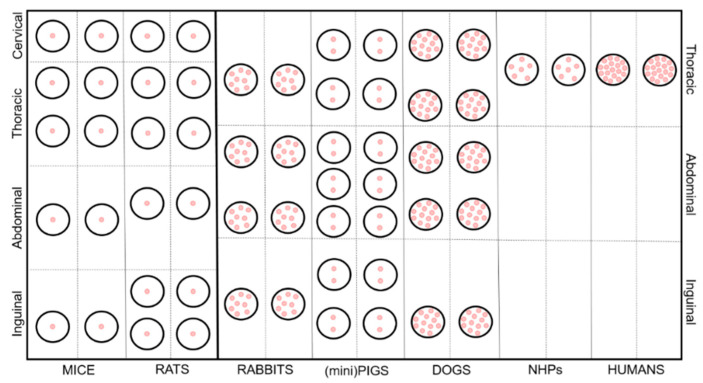
Schematic representation of the anatomical features and distribution of the mammary gland in the analyzed species. Pink dots represent the canals/ducts.

In general, mammary glands can be defined as modified glands that give name to the Mammalia class, whose secretion is vital for offspring survival. They are complex tubule-alveolar glands made of secretory units organized into lobules, surrounded by connective tissue septa [9]. From a developmental point of view, they originate as epithelial buds growing into the mesenchyme starting from linear ectodermal thickenings, also known as mammary ridges, and subsequently placodes [10]. Mesenchyme starts proliferating around such buds to create a teat/nipple on the skin surface. At this stage, epidermal sprouts start developing, from the buds to the teat/nipple, creating canals. Every canal will then create a separate duct that associates with a glandular mass and a separate orifice [9]. The number of overall glands, teats/nipples and canals vary amongst mammals as shown in Table 1, as well as the anatomical location of the mammary unit, represented in Figure 1. The evolution of the reproductive strategy towards a lower number of newborns, often accompanied by a higher level of maternal care, is the main reason for the large difference between NHP/humans and the other species.

In the analyzed non-clinical species, at birth the gland is just a rudimentary ductal system, that will continue to evolve and grow during puberty and first pregnancy/lactation, under the influence of a wide variety of hormonal factors [10]. As for many developmental patterns, the exact timing of this process varies depending on the mammalian species taken into consideration. As a matter of fact, out of the vast array of mammal tissues, the mammary gland is one of the few undergoing multiple growth, functional development, and regression episodes in the lifespan. With the post-pubertal development, the area of the gland occupied by epithelium increases, with a relative decrease in its stromal component. Such phenomena become even more evident in the late stages of gestation, when alveoli grow, even if, usually, true alveoli are not formed until conception. Generally speaking, it still has to be acknowledged that the majority of critical changes, despite some species-specific differences, occur during pregnancy [10,24]. During gestation, vascularization dramatically increases, and by mid-pregnancy, each alveolus is surrounded by a basket-like network of capillaries [25]. Milk secretion is achieved by the aforementioned alveoli, formed by a single layer of secretory epithelium bound by tight junctions and arranged in a cylindrical manner. Lobules, represented by multiple alveoli surrounded by connective tissue septa, generate lobes upon further bundling [24]. From a histological point of view, mammary glands can indeed be generally described as an epithelial bilayer, within adipose tissue, with two key cell populations: luminal cuboidal cells and star-shaped myoepithelial cells [26,27]. The first population lines the lactiferous ducts, form the inner portion, while myoepithelial cells constitute the outer portion of the bilayer and are responsible for milk ejection [28].

### 2.1. Humans

Humans have a single pair of mammary glands, called breasts, positioned over the *pectoralis major* muscle of the anterior chest. In humans, the mammary tissue is divided into 15–20 lobes of parenchyma separated from each other by a highly variable amount of adipose tissue. Each lobe is drained by its own major lactiferous duct leading to the nipple. The main ducts dilate into small sinuses the closer they are to the *areolus*, where they open directly on the nipple. There are about 11 to 48 minor ducts. It is nonetheless important to acknowledge that the presence of sinuses in the human mammary gland is matter of debate, as pointed out by the work of Ramsay et al. [29]. Surrounding the parenchymal structures are fibrous thickenings of connective tissue, which connect the deep fascia with the dermis of the overlying skin to form a suspensory ligament called Cooper’s ligament. The functional terminal duct lobular unit (TDLU) appears in human breasts upon sexual maturity. According to the area occupied, number of acini, secretory morphology and cellularity, lobular units are classified as Types 1–4, with Type 1 being the least mature and Type 4 lobules being terminally differentiated, milk-producing units found in the lactating mammary gland [11,30].

### 2.2. NHPs

Non-human primates, similarly to humans, have two pectoral mammary glands. The non-lactating mammary gland is macroscopically flattened, but the histologic appearance is nearly identical to human breasts. In macaques, as in women, the mammary tissue lies above and lateral to the nipple, extending to the axilla. The mature gland consists of an arboreous-like ductal system and TDLUs, which are formed of a terminal intralobular duct and surrounding alveoli, embraced by myoepithelial cells. In the non-lactating breast, only approximately 5% of the organ is occupied by glandular epithelial tissue, while the remaining 95% consists of fat, fibrous connective tissue, and vascular and nervous structures. In NHPs, each nipple is crossed by five to seven lactiferous ducts, with varying degrees of communication between the corresponding ductal and lobular units. There are occasional small clusters of glandular tissue in the nipple [22].

### 2.3. Pigs and Minipigs

Out of the analyzed species, the pig shows the highest variability in the number of mammary glands, mainly imputable by the wide range of breeds spread throughout the world. Breeds with higher number of teats have been selected by the farm industry as capable of nursing larger litter sizes, with higher economic profits. Generally speaking, pigs have six/seven pairs of mammary glands, located between the thoracic (two pairs), the abdominal (three pairs) and the inguinal (two pairs) area. Each nipple has two ducts which separately lead to two external openings [24]. At birth, each mammary gland of the piglet is composed of the teat including its thick connective tissue base, an organized fat pad of adipose lobules and connective tissue, two lactiferous ducts, and a few ducts branching into the fat pad. These structures continue to grow until puberty. A significant increase in TDLU development occurs during pregnancy, particularly after day 75; during this period, parenchymal tissue mass increases by over 200%, while parenchymal lipid decreases by nearly 70% [31]. In such a scenario, prolactin has been proven to be responsible for growth and differentiation on porcine mammary epithelium, with late gestational hyperprolactinemia leading to enhanced lactogenesis and milk production without altering vascular development [32,33].

### 2.4. Dogs

Regarding the canine species, mammary glands are arranged into two lines along the ventral surface as for the other species, with two thoracic, one abdominal, and two inguinal pairs. However, the number of the mammary glands in dogs can vary. Indeed, sometimes, the abdominal pair are missing, and occasionally there are more than five pairs. The adult mammary tissue is unevenly divided: the caudal glands are larger and the tissue of the two most caudal glands is usually continuous [19]. The amount of mammary and adipose tissue present is very variable and is more abundant in the abdominal and inguinal glands than in the thoracic glands. Each teat has between 7 and 16 duct openings, and each of these ducts will eventually form a lobe of the adult gland. The epithelial component of the mammary gland is supported by mesenchymal tissue; this includes fibrous connective tissue, adipose tissue, blood vessels, nerves, and lymphatics. As in humans, the fibrous connective tissue may be subdivided into two components: the intralobular component that surrounds the intralobular ducts, and the interlobular component that separates the lobules. The former consists of finer collagen fibers surrounded by a more extensive extracellular matrix, while the latter has larger collagen fibers with less of an extracellular matrix [16]. In dogs, the involution of the mammary glands starts around the eighth week of lactation, and progresses until the end of the third month [34].

### 2.5. Mice

Mice have five pairs of bilaterally symmetrical mammary glands, located along the ventral milk line between the cervical and inguinal area. Such lines can be divided into the cervical–thoracic area, containing three glands on each side, and the abdominal–inguinal region, with two glands on each side. Each gland terminates into a single collecting duct that releases milk through a single teat. Mouse mammary glands, just like rats and rodents in general, do not have separate lobes and are made of a single complex arboreous system. Each rodent mammary gland contains 5–10 secondary collecting ducts, which drain into a single lactiferous duct in the nipple [11]. The mature secretory glandular unit is lobuloalveolar (LA), which undergoes complete maturation only during pregnancy and does not normally persist following weaning. During puberty, terminal end buds start forming, directing ductal elongation. The murine ductal system is primarily surrounded by adipose tissue, with poor fibrous tissue.

### 2.6. Rats

Rats have twelve mammary glands, distributed in six pairs along the milk line, with one pair located in the cervical, two in the thoracic, one in abdominal and two in the inguinal regions. The organization of major lactiferous ducts is similar to the mouse, with a single duct leading to the nipple’s ostia [11]. The mammary glands of females, comprising scattered tubular ducts and alveolar structures, are characterized as tubuloalveolar. There are larger, more contiguous, lobular groups of cells distinguishable for their lack of tubular/ductal orientation [35]. The mammary gland has a compound of branching tubular ducts which terminate in secretory glandular alveoli, also called acini. Lobules are composed of groups of alveoli. As for the rat and the human species, the basic milk-producing unit is the TDLU, composed of a lobule associated with intralobular and extralobular terminal ducts [11].

### 2.7. Rabbits

The number of mammary glands in this species can vary from 8 to 10 depending on the genetics of the animals [18], with approximately 6–7 ductal systems per gland [36]. They are distributed from the ventral thoracic to the inguinal regions: two pairs of thoracic, two pairs of abdominal and one pair of inguinal mammary glands. Each nipple has about 8–10 ostia [24]. The presence of sinus-like dilatation of the ducts has been described in pregnant/lactating European breeds, seemingly acting as a milk reservoir [36]. In this species, the majority of the mammary developmental process takes place during gestation (approximately 67%), with the remaining 33% occurring during lactation [37,38]. It is nonetheless important to mention that growth dynamics seems to be influenced both by breed, husbandry conditions, and experimental methodologies employed [37].

## 3. Physiology of Lactation

Lactation can be defined as the process that combines milk secretion and its removal and represents the final stage of the reproductive cycle. In order for it to be successful, three pivotal events have to occur: proliferation of alveolar epithelial cells, their structural and biochemical differentiation and, finally, synthesis and secretion of milk [24]. The process that leads to milk production is also known as lactogenesis and is critically linked to the acquisition of secretory capabilities by mammary alveolar cells. It is commonly divided into lactogenesis I, II and III [39]. Lactogenesis I is also referred to as “secretory differentiation”, while lactogenesis II is “secretory activation” [40]. During lactogenesis I, mammary epithelial cells (MECs) undergo morphological differentiation and become competent to produce and secrete some milk components referred to as colostrum [41]. In such phases, production of milk components seems to be restricted to a limited number of alveolar MEC because some secretory mechanisms are still incomplete [39]. During these late phases of pregnancy, milk production is blocked by the high levels of estrogens and, most importantly, progesterone, a steroid hormone also known as the “pregnancy hormone” because all mammals rely on this hormone to maintain pregnancy. Despite differences amongst species in progesterone production during pregnancy, a drastic drop in its production at parturition is always present [42]. Such a drop allows for the initiation of the lactogenic complex activation and milk production, also referred to as lactogenesis II [24]. Indeed, high levels of progesterone are capable of inhibiting the most pivotal hormone related to lactation: prolactin (PRL). Its circulating concentrations slowly increase during pregnancy so that, by the end of gestation, levels are up to 20-fold higher than pre-pregnancy reference values. Upon the clearance of progesterone and estrogens at parturition, PRL can start promoting the transcription of casein mRNA, stimulating the synthesis of α-lactalbumin, and increasing lipoprotein lipase activity in the mammary gland [43]. It is extremely important to acknowledge that PRL production, distribution, and its physiological functions are quite different in rodents when compared to humans and other mammals [44]. Once initiated, milk secretion continues but with variable rates over time [24], as is referred to as lactogenesis III [45]. Removal of the milk from the mammary gland is necessary to maintain its production and secretion. The overall control of milk secretion requires a strong interaction between both physical and chemical factors. Concerning physical factors, most important is the pressure exerted from the milk present in the alveoli that leads to an inverse relationship between milk production and intra-mammary pressure. As milk builds up within the mammary gland, crucial supporting structures such as blood vessels are displaced, resulting in poor delivery of nutrients to the alveolar cells. Once milk is removed from the gland, pressure drops, and then slowly starts building up again as new milk is produced [24]. On the other hand, chemical control of milk production occurs locally by means of an autocrine protein fraction produced by MEC known as the feedback inhibitor of lactation (FIL) [39]. Currently, the exact mechanism of action of FIL is not completely clear, but it seems to be capable of slowing down milk production by suppressing key factors, stimulating the intracellular breakdown of casein, reducing the number of PRL receptors, and inhibiting MEC differentiation [24]. Finally, another key hormonal factor in the lactation process is oxytocin. Suckling or manual stimulation of the teat is locally detected, and the stimulus is transmitted by sensory afferents to the hypothalamus, which then initiates oxytocin release from the neurohypophysis. This hormone stimulates the myoepithelial cells that surround the alveoli to contract and cause milk to flow from the alveoli through the duct system to the teat end [28,46]. The physiology of lactation does not differ drastically amongst species, but the duration and the yield of both colostrum and milk are highly variable (Table 2).

### 3.1. Humans

Oxytocin, which slowly increases during late gestation and peaks at parturition, triggers milk ejection by inducing the contraction of myoepithelial cells and possibly, by direct effects on the secretory activity of MEC, even bonding and maternal behaviors are regulated by oxytocin. In women, little to no milk can be obtained without activation of the milk ejection reflex (activation of both oxytocin and PRL release). To be consistent with the duration of lactation in other primates, the average duration of lactation in women in ancient times would be expected to be about 3–4 years [59], but nowadays the duration of breast-feeding in traditional highly industrialized societies varies greatly. It is impossible to determine “normal” weaning behavior for both women and other mammals because the artificial termination of the lactation period is based either on social and cultural “acceptability” or economic expediency [49].

### 3.2. NHPs

Non-human primate species have a high degree of similarities with humans. Oxytocin is low during the third trimester of pregnancy, peaks on the date of parturition, and returns to baseline levels during lactation [57]. Prolactin is not an obligate component of mammary growth and development in macaques but is required for lactation; this hormone is not as strong of a mitogen in the NHP breast as steroid hormones or growth hormone (GH). In both human and non-human primates, the hepatic and intra-mammary enzymatic systems are present for the conversion of precursors to a more bioactive estradiol (aromatase and steroid sulfatases); thus, the amount of local estrogen exposure in the breast correlates only weakly with the serum concentration. Gestation in macaques is approximately 150 days in length, and during this time, the breast, as in other mammals, undergoes extensive growth and differentiation under the influence of high systemic concentrations of estrogens, progestogens, chorionic gonadotropin, placental lactogen, and PRL. The change in volume of the glandular tissue is roughly ten-fold to twenty-fold, as a result of both epithelial proliferation and secretory distention of the ductal and alveolar system [22].

### 3.3. Pigs and Minipigs

The peripartum PRL surge begins about two days prepartum and extends through several days postpartum, although it remains significantly greater than those found during most of pregnancy. The prepartum peak of PRL is essential for the onset of lactation, and the decline slowly begins over the initial days postpartum [49]. PRL has indeed been proven to direct growth/differentiation of epithelial cells in the mammary gland throughout gestation, with an experimentally induced late gestational hyperprolactinemia being responsible for higher milk yields [32]. In pigs and minipigs, the hormone relaxin from the corpora lutea has a similar function to placental lactogen, produced in humans and rodents, which is a PRL agonist [42,60]. Regarding the porcine species, most of the studies concerning lactation have been carried out under intensive breeding conditions; there is little information about pigs’ lactation in wildlife. Wild sows build a nest for their litters, and the piglets remain in the nests for about two weeks and are weaned after an eight-week lactation. In commercial intensive piggeries, the length of lactation has been truncated to 21–28 days (3–4 weeks), to increase profitability. The important role of milk ejection in lactation is clearly illustrated by the characteristic behavior pattern associated with suckling and the oxytocin release in the domestic sow, resembling what happens in humans. The piglets jostling and nuzzling on the mammary glands induce oxytocin release, followed by rapid ejection of milk from the mammary glands. Piglets have a very short amount of time, in terms of minutes, to obtain all the possible milk from their preferred nipple [49].

### 3.4. Dogs

Dogs also show a similar physiology of lactation to humans, with the drop in progesterone and estrogen post-parturition, and increased levels of PRL from mid-gestation to weaning, with higher spikes starting at parturition. Placental relaxin secretion starts from mid-gestation, decreases in the third trimester, and drops at parturition [61].

### 3.5. Mice

Although progesterone signaling controls alveolar proliferation, PRL directly controls epithelial cell differentiation [62]. Its release is essential for the proliferation and functional differentiation of lobulo–alveolar structures during pregnancy [63]. Moreover, as opposed to humans, PRL has a strong luteotropic action in rodents, promoting progesterone production during pregnancy [44]. The estrogen hormone has receptors in both stromal and epithelial cells, but it is required only in the stroma for proper ductal development. Oxytocin, when released, induces the contraction of the myoepithelial cells surrounding the alveoli and thereby induces milk ejection. Thus, oxytocin is not only necessary for postpartum milk ejection but also for alveolar cell proliferation [62].

### 3.6. Rats

The physiology of rat lactation is similar to that reported for the mouse. In rats’ mammary glands, PRLR expression is low during most of pregnancy and starts increasing on day 21, potentially in response to the pre-partum rise in pituitary PRL release, and continues to increase throughout lactation [64]. In female rats, lactation induces the mobilization of fat stores and a large increase in food intake, depending on the size of the suckling litter. In rats, PRL secretion is suppressed during the second half of pregnancy [65]. The placenta produces the placental lactogen, which binds to PRL receptors and stimulates growth and differentiation of epithelial cells in the glands in the same way as PRL [60].

### 3.7. Rabbits

Data regarding the physiology of lactation in rabbits are unfortunately relatively poor. Nonetheless, the interest toward this species as a potential model for breast cancer has led to some interesting studies and updated reviews [37]. What is known is that lactation usually lasts up to 4–5 weeks [49], and that the litter only suckles once or twice a day. Additionally, in this species, the key factor for lactation and its maintenance is PRL [50].

## 4. Colostrum and Milk Composition

Milk and colostrum composition vary greatly among animal species, as reported by a previous review of the literature [66]. Components of milk and colostrum include proteins, lipids, carbohydrates, minerals, vitamins, and cells. The milk components were found to be influenced considerably by the stage of lactation, where these changes differ often from one species to another. In general, colostrum differentiates from milk mainly due to its high immunoglobulins (IgG) concentration. It is important to acknowledge that the different types of placentation and active immunity transport mechanisms, peculiar to each species, highly influence the degree of the colostrum-mediated transfer of immunity. The degree and timing of immunity transfer during pregnancy to the offspring impacts on the importance of colostrum for immunity transfer [24,67]. In humans and NHPs, IgG are transferred to the fetus during the second and the third trimester of pregnancy, while in rodents and rabbits this occurs mainly during organogenesis [67,68]. In other mammalian species, such as dogs and pigs, IgG transfer during pregnancy is minimal; hence, the immunity transfer from the dam to the offspring is essentially lactogenic, with 85–95% of the blood immunoglobulins originating from colostrum transfer [69,70]. Overall, IgG colostrum concentration is specifically high after parturition and rapidly drops. In addition to systemic immune protection, colostrum also plays a major role for the local digestive system due to the presence of IgA, isoenzymes, lactoferrin, white blood cells, and various cytokines. The newborn absorbs colostrum IgG from the digestive tract into the blood stream. The newborns’ ability to absorb IgG ends shortly after parturition [51]. Although colostrum has higher concentrations of immunoglobulins in all mammalian species, the concentration of other components may vary between species. Very few papers investigating the milk composition early in lactation were found in our search. In addition, different components and methods were investigated and used at each paper. The main characteristics and differences of the mammary secretion of the species examined for colostrum and “mature” milk are shown in Table 3 and Table 4, respectively.

### 4.1. Humans

As for the other mammals, the composition of breast milk is affected by different factors and, depending on the individual, changes over the course of a single breast-feeding session, of a day, and through lactation [77,89]. One of the key factors seems to be represented by the maternal diet: indeed, dietary intake of different nutrients, particularly fatty acids and some micronutrients, is related to their content in breast milk composition, but does not affect macro-nutrient composition [91]. Generally speaking, fat concentrations tend to increase not only over the course of a day, but also during the same breastfeeding session [89]. On the other hand, the overall protein and amino acid contents show a marked decreasing trend over time during the first year of lactation [77]. One of the few components that seems to remain relatively stable for the entire duration of lactation, except for colostrum, is lactose [77]. Regarding minerals, magnesium, phosphorus, and calcium show significant lactation-stage-specific differences and high inter-individual variations, while manganese, copper, and iron remain relatively constant during all lactation stages. Zinc concentrations, overall, show a decreasing trend as lactation progresses [77].

### 4.2. NHPs

Non-human primate milk is relatively diluted: it generally consists of <15% dry matter, with about 7% sugar, ≅3–6% fat, and ≅1–2% proteins, and changes over the course of lactation. It is important to acknowledge that, amongst different NHP species, great differences in milk composition can be observed, mainly imputable to the length of lactation, frequency of feeding, and milk yield [92,93]. Most studies investigating the NHP milk composition used the rhesus macaque, while few analyses of the milk composition of cynomolgus monkeys, which is the most common NHP in research, were found. In rhesus macaques (*Macaca mulatta*), both fat and protein contents increase as infants age, in the light of the higher demand in energy. Such increase in energy content seems to be related to lower milk yields. Out of the different components, fat percentages show the highest inter-individual variation within the same species, while lactose levels are relatively stable [86]. However, differences in milk composition among prosimians may be related to differences in maternal care: species that carry their offspring produce more dilute milk, with higher yields, when compared to species that usually leave newborns for prolonged period. Lorises, bushbabies, and potentially cheirogaleids produce relatively rich, energy-dense milks in comparison with anthropoid primates, such as rhesus macaques (*Macaca mulatta*), white handed gibbons (*Hylobates lar*) and gorillas (*Gorilla gorilla gorilla*) [92].

### 4.3. Pigs and Minipigs

When compared to mature milk, colostrum has higher concentrations of protein, particularly immunoglobulins, some minerals (particularly copper, iron, iodine, and zinc) and vitamins, hormones, and growth factors. Lactose is present in lower concentrations in colostrum than in mature milk. Milk fat concentration transiently increases during the period from day 2 to day 4. The composition of milk after approximately day 7 to day 10 is relatively stable for the remainder of lactation. As for other species, maternal diet can affect some milk components, including concentrations of fat, fat-soluble vitamins, and some minerals, as well as proportions of specific fatty acids. Some components of sow milk also are affected by genetics, parity, colostrum and milk yield, and ambient temperature [56].

### 4.4. Dogs

Early studies showed that dog milk composition might change with breed. For this summary, we focused on the beagle dog because it is the commonly used breed for research purposes. To summarize, the concentration of iron, zinc, calcium, protein, and fat showed patterns that were influenced by the stage of lactation. The concentration of copper, manganese, magnesium, and carbohydrates were not significantly affected. Adkins et al. [73] found that protein concentration was high in colostrum, decreasing significantly by day 21, and then slightly increased throughout the duration of lactation. This pattern of decreasing protein concentration during lactation is similar to humans. However, Lönnerdal et al. [82] reported that protein concentration increased over time. As for humans, a decreasing pattern in the concentration of all amino acids with the increasing lactation stage was observed. The lipid content does not show remarkable changes during the lactation period. Slight non-significant decreases were observed between days 14 to 28. The lipid concentration in dog milk was higher than that reported in humans [73]. Lactose levels were low in colostrum, increasing gradually until day 28, followed by a slight decrease. Iron concentration increased significantly from day 1 to day 3, and then gradually decreased by day 42. This is in contrast to humans, where iron concentration is high in the colostrum and then gradually decrease during lactation [73]. Lönnerdal et al. [82] reported that the zinc concentration decreased throughout lactation, while Adkins et al. [73] reported that zinc concentration slowly increased from day 1 to day 14, and then decreased by day 42. Zinc levels were higher than those reported for humans [73,82]. Copper concentration was slightly higher in early lactation and then gradually decreased throughout lactation or remained unchanged. Copper levels were generally higher than those reported in humans. Milk calcium concentration was lower in colostrum but increased thereafter, peaking on day 35. Calcium levels were significantly higher than those reported for humans [73,82]. Magnesium concentration was highest in the colostrum but rapidly decreased by day 3 and remained relatively constant during lactation. Concentration of phosphorus showed a very mild increase from day 3 to day 28 [73]. The iron concentration in the dog milk is influenced by the stage of lactation, with values decreasing over time. This is similar to what was reported in other species; however, the iron concentration in dog milk was found to be considerably higher than that of human milk. The manganese concentration was not found to be influenced by the stage of lactation, and its levels were higher than reported for human milk. The fat content of canine milk was found to be influenced by the stage of lactation, with concentrations increasing during the first part and decreasing during the last part [82]. The level of carbohydrates was fairly constant and did not show a strong developmental pattern.

### 4.5. Mice

Very few papers have been published on mouse milk composition. The composition of mouse milk can vary considerably between mouse strains. In general, the analysis on mouse milk is challenging due to the small sample volume and the high fat content. Hopefully, the improvements of new analytical methodologies should fill in our knowledge gaps, as suggested by the work of Stewart and Davis [28]. Crude protein levels did not change during lactation [48,94,95]. Crude fat increased from day 3 of lactation to day 14 and remain stable until day 18 [48,94], while in another study the fat content decreased from early to mid-lactation [96]. Lactose content increased with lactation day [48,95]. Great variability was noted in lactose content and crude protein between strains of mice.

### 4.6. Rats

There are characteristic differences in the nutrient content of milk among strains of rats, particularly for changes in the lactose and fat content of the milk during the lactation period. In general, rat milk elements (iron, copper, zinc, manganese) show a similar pattern of high initial levels, mainly during the colostrum phase, that decreases throughout lactation. The concentration of some elements increases during the last phase of lactation (iron, manganese). The iron concentration of rat milk, considerably higher than that found in human milk and much greater than its plasma levels, rapidly decreases during the first part of lactation (approx. 40% drop). Thereafter, it continues to decrease but in a less pronounced manner and increases in the last days of lactation (days 25–28). The decreasing pattern was also found in humans, although the percentage decrease is not that high. Colostrum copper concentration is considerably higher than that of humans and decreases until day 11; it then remains relatively unchanged until the end of lactation. The change in copper concentration pattern is similar to that observed in humans, however the copper concentration in rats’ milk is much higher. The same applies to zinc, showing a similar pattern to humans despite higher overall levels. The concentration of manganese decreases significantly from day 0 to day 12 and remains low, but increases to nearly initial levels by the latter days of lactation. A similar pattern of manganese concentration was reported in humans. In contrast to other elements, the concentration of manganese in rat milk is not considerably higher than that of humans. Magnesium concentration was fairly stable during early and mid-lactation and decreases during late lactation. The protein and calcium concentration increase steadily during lactation until day 24 and decreases at the end of the lactation period. The similar patterns of calcium and protein is likely due to the fact that the major protein of rat milk is casein, which is well known for its calcium binding capability [97]. In contrast to rats, human calcium levels decline during lactation. Carbohydrate concentration increases during the first half of lactation, then decreases during the second half. In humans’ milk, the carbohydrate level is much higher than in rats. In humans an increase in milk carbohydrate is also found in the early lactation period, but there is no decrease at later stages of lactation. While lactose is by far the major carbohydrate in human milk, rat milk may contain significant amounts of neuraminlactose. Quantitatively, the most important constituent in rat milk is fat. Similar to humans, the fat content of the rat milk did not exhibit a strong pattern during the lactation period [71].

### 4.7. Rabbits

Kits are weaned at the age of 4–5 weeks and are exclusively dependent on milk until lactation day 18–19. Rabbit milk yield corresponds to kits’ weaning stage and reaches its peak around lactation days 17–21. It is important to remember that rabbits only nurse kits once or twice a day, strongly impacting the nutritional value of the milk. In general, rabbits’ milk is concentrated with fat, protein, and energy, but nearly absent of lactose, and the composition does not vary significantly between most breeds [72]. As in other mammalian species, the colostrum has higher protein content due to high immunoglobulin levels. This increases the dry matter value of colostrum relative to rabbit milk. Protein content decreases along with the increase in milk yield during lactation. Apart from protein content, the composition of rabbit milk is quite stable during the second and third weeks of lactation. The changes in composition in the later stage of lactation are closely related with the decrease in milk yield. Mineral element composition changes substantially after the lactation peak. In general, rabbit milk is rich with calcium, sodium, and potassium. Calcium concentration, and to a lesser extent phosphorus, increases with the progressing lactation stage, while the effect on potassium and sodium is less clear and there are different data values reported in different papers. Magnesium content increases with lactation stage, while zinc, copper, iron, and manganese decrease gradually in concentration as lactation progresses [72].

## 5. Discussion

### 5.1. Anatomy of the Mammary Gland

As expected, from a gross anatomy point of view, the variations between the analyzed species are very high. When looking at the number and the position of glands, NHPs better resemble the human situation, with differences only in the ducts. The number of teats is directly related to the number of the offspring [98]; therefore, such a situation was to be expected. Pigs are on the opposite side of the spectrum because, depending on the breed and in light of the pork production requests, sows can deliver up to 18 piglets. The human mammary gland, out of those taken into account, is the one with the highest number of canal/ducts, followed by the canine species. A peculiar situation can be noted in rodents, where only one canal per teat is present. When selecting a relevant animal model for trials involving lactation, the similarity in gross anatomy is not necessarily one of the key decisional factors. Indeed, having a higher number of mammary glands and teats often allows for easier sampling procedures and, potentially, higher volume specimens.

### 5.2. Physiology of Lactation

No major discrepancies were noted regarding the physiology of lactation amongst the analyzed animals. Indeed, hormonal inputs and pathways are pretty much conserved, with PRL and oxytocin being pivotal. Nonetheless, PRL seems to play different roles in rodents when compared to the other species analyzed in the present literature review. When looking at the most important veterinary textbooks for such topics, the bovine species is always the most included and analyzed within the physiology of lactation chapters, because its role in the milk industry for human consumption is undeniable. This represents a relative limitation for experimental animals, and in particular laboratory animals, because the peculiar gastrointestinal apparatus of ruminants changes the metabolic scenario of milk production. Several knowledge gaps need to be filled regarding the physiology of lactation, both at systemic and molecular levels, including in-depth characterization of hormonal mechanisms of action and environmental influences, among others.

### 5.3. Colostrum and Milk Composition

The comparison of colostrum/milk compositions between the different species can be hard to interpret in light of several technical and physiological issues. Indeed, despite representing the basis of today’s knowledge, a lot of the retrieved papers are relatively old, often relying on low sample sizes and less accurate analytical tools when compared to modern analyses. In addition, different components and methods were investigated and used in each paper. Moreover, high inter-individual variations, mainly qualitative, within the same species are extremely common, and depend on a wide variety of factors including maternal nutritional status and diet, number of newborns, or duration of lactation. Colostrum of all species contains high levels of IgG and other immune factors which increase the protein content. The level of IgG decreases within a few days post-partum. As for the other components of both milk and colostrum, they vary between species and the NHPs seem to better resemble humans’ mammary gland secretions, which are definitely more diluted when compared to other species such as rabbits, rats and mice. This finding was to be expected because, as already mentioned, the number of newborns and maternal care are amongst the key factors. Nonetheless, (mini)pigs show a relatively close similarity with humans’ milk gross composition, despite their capability to produce high litter sizes.

### 5.4. Practical Considerations

Choosing an animal model for a particular trial is always difficult and often implies the necessity to cope with knowledge and information gaps. The topics covered here represent the starting point for a conscious decision, because anatomical and physiological similarities to humans are the basis of a high translational experimentation value. Looking at the results, NHPs, as expected, seem to represent the best model to enroll in studies regarding lactation. Nonetheless, other factors have to be considered. In the last decade, the scientific community has come to the agreement of using experimental NHPs only when strictly necessary [99]. Moreover, trials involving NHPs are expensive, long, and due to ethical considerations have low sample sizes that can undermine the outcome data. Finally, collecting milk samples from such species could be extremely difficult in light of the maternal behavior. Ease of sample collection should always be an important factor when designing animal trials, because choosing a “difficult” species may lead to failure, especially in “longer” trials that require un-sedated animals and/or repeated samples. Smaller animals such as rodents and rabbit are not necessarily the best choice for lactation studies. Indeed, in order to collect milk from rodents, one of the few options is the euthanasia of pups to collect gastric content right after suckling. Other methods, such as using mini-pumps to collect milk directly from the mother, often require pharmacological support to increase milk volume, a strong bias in drug lactation transfer studies, and still leads to small volume of samples. Furthermore, when compared to humans, their milk composition was quite different and discrepancies in PRL production and functions were highlighted. In such a scenario, larger animals such as dogs and pigs seem to be a good fit for lactation trials. With regard to dogs, it is important to acknowledge that, despite their relevance for regulatory toxicology studies, their use for biomedical research often raises strong criticism and ethical issues in public opinion, especially in Europe. On the other hand, the enrolment of pigs in research trials seems to be more widely accepted and is considered as a valid alternative when anatomical/physiological differences are not relevant [100]. Minipigs, in particular, offer all the advantages of conventional pigs including genetic and metabolic similarities to humans, avoiding the main problem represented by the size. Their use in the biomedical setting is well established and recognized, and the availability of in-depth physiological characterization and the related physiology-based models, vital for results interpretation, is increasing. Moreover, Göttingen Minipigs are specifically produced for biomedical purposes, with a high standardized genetic background and health status.

## 6. Conclusions

In conclusion, the present analysis of the literature confirmed the complexity of the decisional process behind the choice of an animal model for in vivo trials. For some of the reviewed species, data were either poor or completely missing, highlighting the necessity to generate more physiological background studies for species that are routinely used in laboratory settings. Overall, pigs, and in particular minipigs, seem to represent the better choice when looking at both physiological similarities with humans and the feasibility of lactation trials.

## Figures and Tables

**Table 1:** Anatomical features of the udders of the species taken into account; pr = per row.

Species	Number of Glands	Number of Teats/Nipples	Number of Canals per Gland	Location	References
Rats	12	12	1	cervical (1 pr) thoracic/pectoral (2 pr) abdominal (1 pr) inguinal (2 pr)	[11,12]
Mice	10	10	1	cervical (1 pr) thoracic/pectoral (2 pr) abdominal (1 pr) inguinal (1 pr)	[11,13,14,15,16]
Rabbits	8–10	8–10	6–10	thoracic (1 pr) abdominal (2 pr) inguinal (1 pr)	[17,18]
Dogs	8–10	8–10	7–16	thoracic (2 pr) abdominal/inguinal (2–3 pr)	[16,19]
(Mini)Pigs	10–18	12–18	1–3	thoracic (2 pr) abdominal (3 pr) inguinal (2 pr)	[20,21]
Non-Human Primates (NHPs)	2	2	5–7	pectoral (1 pr)	[22]
Humans	2	2	10–25	pectoral (1 pr)	[11,23]

**Table 2:** Duration and yield of colostrum and milk production.

Species	Duration of Colostrum Production	Yield of Colostrum	Duration of Milk Production	Yield of Milk	References
Rats	/	/	~21 days	/	[47]
Mice	/	/	~18 days	0.1–0.5 mL	[48]
Rabbits	/	/	4–5 weeks	100–200 g/day	[49,50]
Dogs	48 h	270 mL/day	~8–10 weeks	~1000 mL/day	[51,52,53,54]
(mini)Pigs	24 h	~3.75 kg/day *	~8 weeks	4500–5700 g/day *	[55,56]
NHPs	/	/	~12 months	/	[22,57]
Humans	96 h	~500 mL/day	~6 months	~800 mL/day	[39,58]

/, data not available; *, these data only refer to standard pigs.

**Table 3:** Colostrum composition.

Species	Dry Matter %	Protein %	Casein %	Whey Protein %	Fat %	Lactose %	Fe µg/mL	Cu µg/mL	Zn µg/mL	Mn µg/mL	Mg µg/mL	Ca µg/mL	P µg/mL	References
Rats	/	8.6–9.1	/	/	13.6–15.7	2.3–2.6	8.1–9.2	8.6–9.8	13.3–14.2	0.3–0.4	168–180	755–829	/	[71]
Mice	/	/	/	/	/	/	/	/	/	/	/	/	/	/
Rabbits	31.4–33.7	13.5–15.9	/	/	13.7–20.4	1.6–2.1	/	/	/	/	/	/	/	[72]
Dogs	/	12.4–16.2	60.7	39.3	13.2	1.7	3.7	1.3	5	/	128	1363	935	[73]
(mini)Pigs	20.1–26.7	7.7–16.6	1.5–3.4	7.9–14.8	6.4–8	2.8–3.9	/	/	/	/	100	800	1000	[56,74]
NHPs	/	2.2–2.7	/	/	4.3–6.3	7.7–7.9	0.9–2.6	2–4.1	3.5–6.8	/	37.5–61.7	324–347	/	[75]
Humans	11.92	2.6	0.4	1.18	3	5.8	1.1	0.4	4.8	0.01	32	293	159	[76,77,78]

Data are expressed as ranges or single values; /, data not available.

**Table 4:** Mature milk composition.

Species	Dry Matter %	Protein %	Casein %	Whey Protein %	Fat %	Lactose %	Fe µg/mL	Cu µg/mL	Zn µg/mL	Mn µg/mL	Mg µg/mL	Ca µg/mL	P µg/mL	References
Rats	27.9–32.8	8.9–9.7	6.4–8	0.9–2.5	14–15.9	1.1–4.1	4–7	1.7–7	9-55	/	158–192	2849– 3206	1600– 2720	[79,80]
Mice	36.3–39.4	10.1–12.7	/	/	19.3–22.9	2.4–2.8	/	/	/	/	/	/	/	[48]
Rabbits	31.2	10.3	/	/	15.2	1.8	0.003 ^§^	0.002 ^§^	0.02–0.03 ^§^	0.0001 ^§^	0.35–0.45 ^§^	2.71–5.36 ^§^	2.44–3.28 ^§^	[72,81]
Dogs	22.7–26	4.3–9.8	65.8–75.4 *	26.4–34.2 *	2.4–13.4	29.3–40.2	1.8–13.1	0.9–2	4.1–9.6	0.1-0.2	55.8–104.3	1366–2440	914–1401	[52,73,81,82,83]
(mini)Pigs	18.8–22.7	5–7.5	2.7–3.6	2.4–5.4	7–10.1	4.3–5.6	/	/	/	/	105	2000	1420	[56,74]
NHPs	12.2–14	1.3–2.3	45 *	55 *	3.3–6.2	4.8–9.1	/	/	/	/	34	380	152	[81,84,85,86,87]
Humans	12.6	1.2	0.3	0.7	4.1	7	0.5–1.8	0.2–5.2	0.7–3.8	0.01-0.03	25–33	230–310	130–190	[88,89,90]

Data are expressed as ranges or single values; /, data not available; *, % on total proteins; ^§^, these data are expressed in g/kg.

## Data Availability

No new data were created or analysed in this study. Data sharing is not applicable to this article.
